# Determinants of dietary behavior and physical activity among Canadian Inuit: a systematic review

**DOI:** 10.1186/s12966-015-0252-y

**Published:** 2015-06-24

**Authors:** Victor O. Akande, Anna M. Hendriks, Robert A. C Ruiter, Stef P. J. Kremers

**Affiliations:** Department of Health Promotion, NUTRIM School for Nutrition, Toxicology and Metabolism, Maastricht University Medical Center, P.O. Box 616, 6200 Maastricht, MD The Netherlands; Department of Health Promotion, CAPHRI, School of Public Health and Primary Care, Maastricht University, Maastricht, The Netherlands; Department of Work & Social Psychology, Faculty of Psychology & Neuroscience, Maastricht University, Universiteitssingel 40, Maastricht, 6200 MD Netherlands

**Keywords:** Canada, Inuit, Dietary, Food, Physical activity, Behaviour, Change, Review

## Abstract

**Background:**

Increased dependence on Western diets and low physical activity have largely contributed to weight gain and associated chronic diseases in the Canadian Inuit population. The purpose of this study was to systematically review factors influencing dietary and physical activity behaviors to guide health promotion interventions and provide recommendations for future studies.

**Method:**

We conducted a systematic literature review to identify relevant articles. Searches were conducted between May 2014 and July 2014, and inclusive of articles published up until July 2014. Articles were searched using four databases: PubMed, PsycINFO, SocINDEX, and Psychology and Behavioral Sciences Collection. Eligible studies focused on diet and/or physical activity or determinants of diet and/or physical activity in Canadian Inuit population, and were published in English.

**Results:**

A total of 45 articles were included in the analysis. A detailed appraisal of the articles suggested that many Inuit have disconnected from the traditional ways of life, including harvesting and processing of traditional food species and the associated physical activity. In the last two decades there has been a significant shift from consumption of healthy traditional foods to energy-dense store-bought foods particularly among younger Inuit (<50 years of age). Additionally, low socioeconomic status (SES) and high transportation cost affect food accessibility and contribute to poor dietary choices in the population. However, a few articles that described the mediating role of psychosocial factors reported that higher SES, increased healthful food knowledge, and self-efficacy towards healthy dietary behavior, were associated with greater intentions to make healthier food choices and participate in physical activity.

**Conclusion:**

It is evident that the rapid social, cultural, and environmental changes in the Arctic have altered dietary and physical activity behaviors of Canadian Inuit. However, our understanding is limited on how these behaviours might be influenced in the face of these changes. Prospective studies are needed to advance our knowledge of cognitive and environmental determinants of Inuit energy balance-related behaviours. These studies can inform the development of health promotion interventions in the population.

## Background

Over the past 50 years Canadian Inuit have been undergoing rapid dietary transition, which reflects a shift away from the traditional ways of living [1]. This shift is largely due to the adoption of Western values and acculturation into Euro-Canadian system. As a result, the population is nowadays developing the so-called ‘energy balance-related’ health problems, such as diabetes and cardiovascular diseases [2, 3]. The rates of overweight and obesity in the population are particularly alarming. According to the 2007–2008 Inuit Health Survey [4], 29 % of Inuit men and 41.6 % of Inuit women are obese. The prevalence of abdominal obesity, measured by waist circumference, is 27.9 % among men and 59.8 % among women, compared to 29.1 % and 40 %, respectively, nationally. An increasing body of evidence suggests that unhealthy behaviors such as the consumption of energy dense food and sedentary lifestyle have resulted in significant weight gain, and are major drivers of the rising rates of diet-sensitive chronic diseases [5–8]. For example, the prevalence of Diabetes Type II was about 1 % in 2002, and had increased to 4.4 % by 2008/2009 [9]. As a result, there is a growing concern among health professionals about the rising rates of obesity, Diabetes Type II, cardiovascular diseases, and certain cancers among Inuit. This has led to increasing calls for more focused health promotion interventions to address the energy balance-related health issues in the population [10–13].

The Inuit are one of the three Indigenous Peoples of Canada and occupy more than one-third of Canada’s total land mass. Canadian Inuit are spread across four regions: Inuvialuit, Nunatsiavut, Nunavik, and Nunavut [14]. “Inuit” in this review includes the Inuvialuit from the Northwest Territories. Nunavut is the largest of the four Inuit regions with approximately 49 % of national Inuit population [14]. The Inuit have the highest population growth rate and are the youngest demographic group in Canada with a median age of 22, compared to 40, nationally [14]. In the 1960s, many Inuit families were relocated to resource-limited environments in the Arctic North by the federal government to protect Canadian interest in the region. As expected, relocation to unfamiliar environment created livelihood challenges for relocated Inuit. For example, the relocation restricted the traditional food [TF] gathering and other cultural activities. Today, Inuit like other Aboriginal groups live and gather foods within spaces which are fractions of the original land mass, and this limits traditional hunting and other food gathering activities [15]. Food gathering was necessary for subsistence, and historically a diet selection process. Healthy TFs were harvested by Inuit on the one hand, and excess energy was lost to food-gathering process on the other hand. Harvesting (hunting, trapping, and fishing) and processing of foods were physically demanding and required some degree of fitness and active living [16, 17].

At present, less local food gathering activities take place among Inuit. There is heavy reliance of households on store-bought processed foods from Southern Canada [18]. Accessibility and availability of healthy foods from the Southern part of Canada are hampered by unique transportation problems. While there are limited road connections among communities in the other Inuit regions and to some southern cities, there are no roads or railways connecting Nunavut to Southern provinces, or between any two Nunavut communities. Air travel is the only means of movement between communities and traveling out of the territory [19], except during summer when sealift activities take place. This results in high living costs, high expenditures on airfares, medical facilities, food supplies, as well as on health programs and services [19]. Although Inuit household income in Nunavut is significantly lower than that of Canadians in other jurisdictions, an average Inuit household in Nunavut however spends twice the Canadian average on food supplies on a monthly basis [20].

Since globalization and its attendant social and environmental changes in the Arctic North cannot be reversed or stopped it is important to explore health promotion interventions for the growing energy balance-related problems among Canadian Inuit. To guide intervention development it is important to identify factors influencing the dietary and physical activity behaviors that have the strongest impact on the energy balance among Canadian Inuit, i.e., the so-called ‘energy balance-related behaviors’ [21]. It is therefore pertinent to identify and systematically analyze behavioral studies that describe dietary and physical activity patterns of Canadian Inuit. Because systematic reviews on this topic are lacking, our objective was to systematically review the literature regarding the socioeconomic, psychological, cultural and environmental determinants of energy balance-related behaviors among Canadian Inuit. Additionally, we systematically assessed the body of literature on intervention studies to determine the strength of evidence and promising practices for the design of effective health promotion intervention programs. This information is of strategic importance to diet- and physical activity-related chronic disease prevention in Inuit population. We aimed to identify gaps in knowledge and propose priority areas for future research.

## Methods

### Search strategy and eligibility criteria

We conducted a systematic review of literature according to the standards described by the Institute of Medicine [22]. The team of reviewers had expertise in systematic reviews. The reviewers conducted the literature searches between May 2014 and July 2014. The team designed a protocol and analytical framework for the entire review process as previously described [22]. Briefly, the topic of interest was formulated and research questions were determined. The search strategy, screening, data extraction process and selection criteria were established. Articles were included up until July 2014. We searched for articles using the key words in two search term strings (Appendix A) in four databases: PubMed, PsycINFO, SocINDEX, and Psychology and Behavioral Sciences Collection. Environmental scan was also conducted to identify relevant grey literatures.

### Inclusion/exclusion criteria

Articles were included only if: research subjects were Canadian Inuit, or articles reported disaggregated subset data of Canadian Inuit in case of multi-jurisdictional studies; if articles examined diet and/or physical activity or other behaviors (e.g., smoking or drinking) in relation to diet and/or physical activity; if articles included determinants of diet and/or physical activity; or examined health promotion interventions to improve diet and/or physical activity. Articles were also included irrespective of year of publication, gender, age, methodology, study design, and if published in English.

Articles were excluded if they: did not report disaggregated data of Canadian Inuit, were multi-jurisdictional; focus was on dietary contaminants or fatty acid profiles; did not report health promotion intervention on diet and/or physical activity. Two authors (V.A. and A.M.H.) independently screened all titles and abstracts of articles that were identified in the literature search for inclusion in the systematic review. Disagreement on manuscript inclusion was resolved by discussion. When in doubt the third author (S.K.) was consulted.

### The review process

**Fig. 1:**
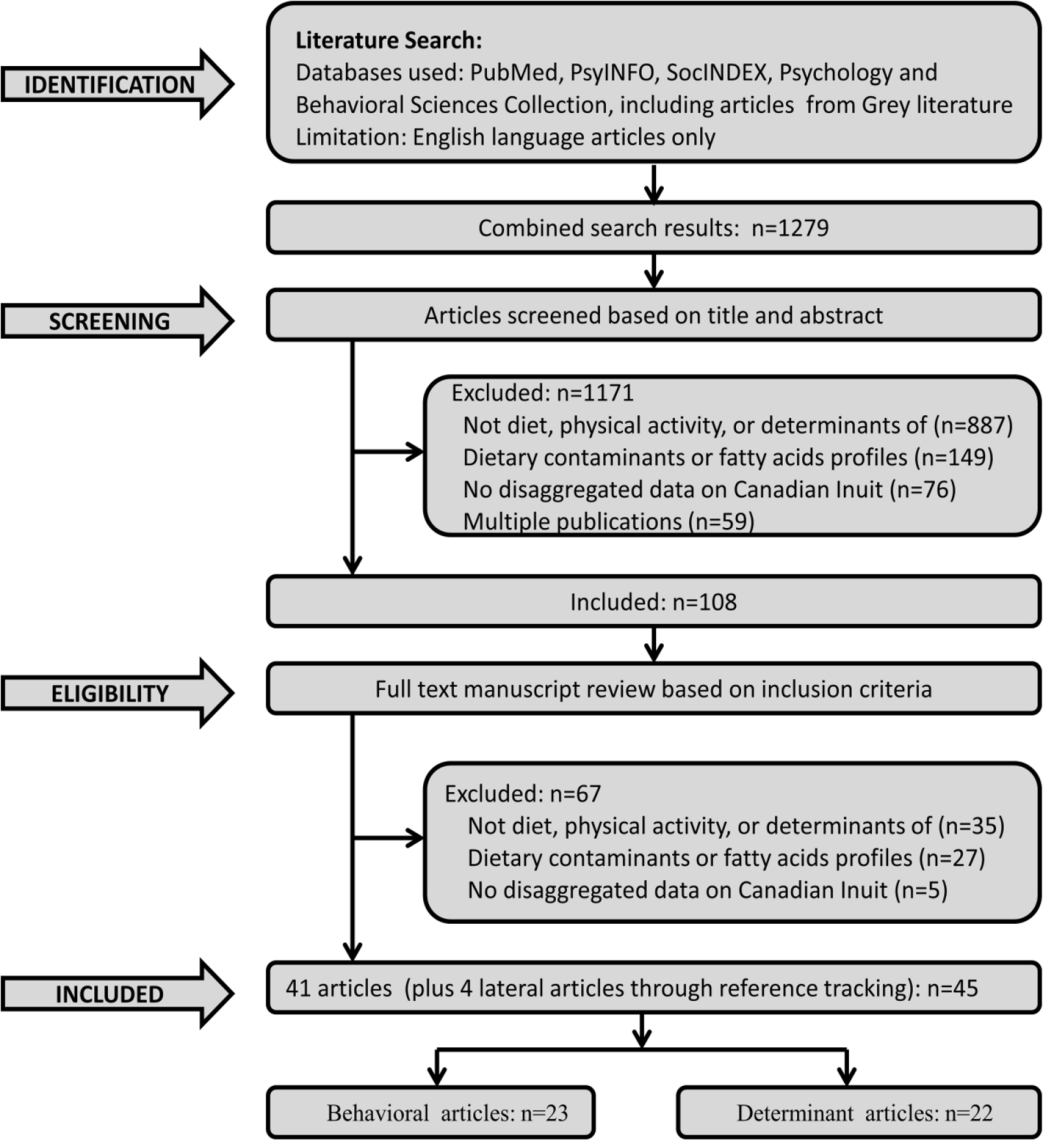
Flowchart of the Systematic Review Process (adapted from the PRISMA process developed by Liberati et al., [23])

The systematic review process (Fig. 1) was divided into four major steps (identification, screening, eligibility, and included) according to the PRISMA scheme; a four-phase reporting procedure for systematic reviews [23]. We started the process with an initial key words search, which produced 1279 records during the identification phase. These records were screened based on title and abstract yielding 108 potential articles for further evaluation to determine their eligibility for inclusion in the study. At the level of title and abstract reviews, a total of 1171 records were excluded from the initial 1279 records. Of these, 887 records were not on diet and/or physical activity, or determinants of either dietary behavior and/or physical activity; 149 articles focused on dietary contaminants and fatty acids profiles; 76 articles did not provide disaggregated data on Canadian Inuit. Additionally, 59 records were eliminated because they were duplicate publications. Full text manuscripts were retrieved for the remaining 108 articles and evaluated to determine their eligibility based on the inclusion/exclusion criteria. Following evaluation, 41 articles were selected for full text review, and a total of 67 publications were discarded. Of these, 35 articles did not focus on diet and/or physical activity, or the determinants of diet and/or physical activity, 27 articles reported on dietary contaminants or fatty acids profiles; and five articles reported no disaggregated data on Canadian Inuit, and were excluded. In addition, lateral article searches using reference tracking produced four additional articles leading to a total of 45 articles for full text review.

### Data extraction and analysis

The 45 articles were thoroughly read. Data were extracted and carefully evaluated using the six phases of thematic analysis described by Braun and Clarke [24]. Briefly, phase 1 – familiarizing ourselves with the data: VA and AM were guided by initial thoughts which were linked to the objectives of the study. We developed search terms (Appendix A) which were used to refine a set of codes for semantic theme analysis. Phase 2 – generating initial codes: VA and AM carefully examined each article for semantic patterns followed by notes taking and coding. The data were then organized into meaningful groupings. The data groups were systematically analyzed for repeated patterns and then coded, and re-coded until linkages were established between groups, and a thematic map emerged. Phase 3 – searching for themes: At this stage, all the relevant coded data extracts were collated systematically and organized into thematic areas. Relationships between codes, between themes, and different levels of themes were established to form overarching themes. Phase 4 – reviewing themes: the identified themes were refined and repeatedly examined for fitness and linkages to associated data. Phase 5 – defining and naming themes: at this stage we defined the identified themes and further refined them, including the scope and contents. We identified two overarching themes at this stage including the corresponding themes. Phase 6 – here we began to write up the results of the thematic analysis, discussion and conclusion.

## Results

The 45 articles that met the inclusion criteria were classified (Table 1) according to methodology (qualitative, quantitative, or mixed), study design (cross-sectional, longitudinal or intervention), and the research participants involved in the study (adult, children or both adults and children). A descriptive summary of each article is provided in Table 2. The 45 articles were systematically evaluated and categorized into two overarching thematic areas: *behavioral* (*n* = 23, 51 %) or *determinant* (*n* = 22, 49 %) in relation to energy balance-related behaviors (Table 1). Behavioral articles were further divided into two themes: dietary behavior and physical activity. We found 18 articles on dietary behaviors and 5 on physical activity behaviors (Table 1). Articles on behavioral *determinants* were classified into six themes: socioeconomic factors (*n* = 9), historical and cultural factors (*n* = 3), smoking and drinking (*n* = 4), health promotion intervention (*n* = 2), climatic factors (*n* = 2), and psychosocial factors (*n* = 2) (Table 1).

**Table 1:** Analysis of 45 included articles based on methodology, study design and emergent themes

Methodology/Design	# of articles (n)	Thematic analysis	# of articles (n)
Research participants		Behavioral articles	23
Adults only (≥ 18 years)	31	Dietary Behavior	18
Children & Adults (≥ 2 years)	9	Physical Activity	5
Children Only (2–17 years)	5	Determinant articles	22
Methodology		Socioeconomic	9
Quantitative	9	Smoking & Drinking	4
Qualitative	6	Historical/Cultural	3
*Mixed	3	Climate Change	2
Study design		Intervention	2
Cross-sectional	40	Psychosocial	2
Longitudinal	3		
Intervention	2		

*refers to a combination of quantitative and qualitative methodologies

**Table 2:** Descriptive summary of 45 articles that met the inclusion criteria for full text review

Study/Authors	Major thematic areas	Sample size	Mean Age ± SD OR age range	Gender male; female	Location	Methology/Design	Major findings
Beaumier & Ford, 2010 [48]	Determinant-Socioeconomic factors	49 participants; 1 community	≥18	Female: (100 %)	Nunavut	Qualitative/Cross-sectional	Education, income, food preferences, climate change, and the absence of full-time hunters in households are barriers to food security
Egeland *et al*., 2010 [33]	Behaviour-Dietary	388 participants; 16 communities	3–5	Male: 47 %; Female: 53 %	Nunavut	Quantitative/Cross-sectional	In Nunavut households 70 % of preschoolers were food insecure, 31 % of preschoolers were moderately food insecure, and 25.1 % experienced *severe* food insecurity.
Egeland *et al*., 2011 [53]	Determinant-Socioeconomic factors	388 participants; 16 communities	3–5	Male: 47 %; Female: 53 %	Nunavut	Quantitaive/Cross-sectional	Children from food insecure households were more likely to have consumed more TFs and less milk compared to children from food secure homes. TF consumption was associated with higher protein and lower carbohydrate intakes and decreased iron deficiency, regardless of household food security status.
Egeland et al., 2011 [20]	Behavior-Dietary	2595 participants; 36 communities	41 ± 14.7	Male: 38 %; Female: 62 %	Nunavut; Inuvialuit; Nunatsiavut	Quantitative/Cross-sectional	The prevalence of food insecurity among adults was 62.6 %. In men, food insecurity was correlated with reduced intake of energy, fibre, Iron, Magnesium, Zinc and vitamin C In women, food insecurity was correlated with higher intake of carbohydrate and lower intake of fibre, folate, vitamins C, and D, Magnesium and Calcium TF consumption was associated with higher intakes of protein, vitamins A and C, lower intakes of carbohydrate, saturated fat, fibre, and Sodium.
Erber *et al*., 2010 [31]	Behaviour-Dietary	64 participants; 1 community	Male: 46 ± 13; Female:45 ± 13	Male: 22 %; Female: 78 %	Inuvialuit	Quantitative/Cross-sectional	The majority of the participants consumed less than their daily requirements of vitamin A while the intake of vitamin D was below recommendations for majority of women. TFs contributed significantly to protein and Iron intake. Store-bought foods particularly juices contributed primarily to carbohydrate and Calcium consumption
Erber *et al*., 2010 [47]	Determinant-Socioeconomic factors	230 participants; 3 communities	Male: 42 ± 14; Female: 45 ± 14	Male: 24 %; Female: 76 %	Inuvialuit	Quantitative/Cross-sectional	Intakes of non-nutrient dense foods were seven times higher than TF consumption. Respondents with higher SES were more likely to consume nutrient-dense foods compared to those with lower SES.
Findlay, 2011 [40]	Behaviour-Physical activity	359 participants;	≥ 12	Male: 56.4 %; Female: 43.6 %	Inuit across Canada	Quantitative/Cross-sectional	There was no significant difference between Inuit and non-Aboriginal respondents who were at least moderately physically active in their leisure time. First Nations respondents who lived off-reserve and Métis were more likely to be physically active than Inuit and non-Aboriginal respondents.
Ford & Beaumier, 2011 [49]	Determinant-Socioeconomic factors	19 participants; 1 community	≥ 18	Not reported	Nunavut	Quantitative/Cross-sectional	Determinants of food insecurity included decreased participation in hunting activities, high cost of traditional harvesting, affordability of store-bought foods, food knowledge and preferences as well as impact of climate change.
Ford *et al*., 2012 [51]	Determinant-Socioeconomic factors	94 participants; I community	≥ 18	Male: 56 %: Female: 44 %	Nunavut	^a^Mixed/Cross-sectional	Users of the community food programs were likely to belong to the lower SES class, unemployed and on social assistance. They were likely to not have hunters in their households.
Gagne *et al*., 2012 [25]	Behaviour-Dietary	217 participants; 10 communities	2 ± 0.88	Male: 52 %; Female: 48 %	Nunavik	Quantitative/Cross-sectional	Although the TF intake was generally low, children who consumed TFs had higher intakes of protein and several micronutrients, and less intakes of energy and carbohydrate, compared to those who did not consume TFs.
Gagne et al., 2013 [62]	Determinant-Intervention	217 participants; 10 communities	2 ± 0.88	Male: 52 %; Female: 48 %	Nunavik	Quantitative/Intervention	Greater proportion of children who participated in the nutrition intervention program met their nutritional requirements of fruits, vegetables, and grains, as well as daily requirements of vitamins, irons, and other micronutrients.
Hopping *et al*., 2010 [13]	Behaviour-Dietary	75 participants; I community	Male: 42 ± 19; Female: 44 ± 16	Male: 9 %; Female; 91 %	Nunavut	Quantitative/Cross-sectional	Dietary intakes of fibre and micronutrients including Calcium, Iron, vitamins A, D, E, were below requirements. TFs were the primary source of protein and Iron while store-bought energy-dense foods were the largest source of fat and carbohydrates in diets.
Hopping et al., 2010 [46]	Determinant-Socioeconomic factors	211 participants; 3 communities	Male: 42.1 ± 15: Female: 42.2 ± 13.2	Male: 17 %; Female: 83 %	Nunavut	Quantitative/Cross-sectional	Respondents who were below 50 years of age consumed non-nutrient dense foods, fruits and vegetables more frequently, and TFs less frequently compared to respondents who were 50 years and over. Respondents with higher education and income were more likely to consume more fruits and vegetables and less TFs.
Hopping *et al*., 2010 [38]	Behaviour-Physical activity	218 participants; 3 communities	Male: 42.3 ± 13.0; Female: 42.4 ± 14.8	Male: 17 %; Female: 83 %	Nunavut	Quantitative/Cross-sectional	Although a large proportion (72 %) of participants was either overweight or obese, 89 % of participants reported moderate to high levels of physical activity.
Hopping *et al*., 2010 [39]	Behaviour-Physical activity	196 participants; 3 communities	Male: 41 ± 14; Female: 45 ± 14.8	Male: 24 %; Female: 76 %	Inuvialuit	Quantitative/Cross-sectional	Although a large proportion (65 %) of participants was either overweight or obese, 89 % of participants reported moderate to high levels of physical activity.
Huet *et al*., 2012 [52]	Determinant-Socioeconomic factors	2595 participants; 36 communities	43.3 ± 0.4	Not reported	Inuvialuit, Nunatsiavut & Nunavut	Quantitative/Cross-sectional	Food insecurity was associated with lower healthy eating index score, intakes of lower vegetables, fruits, grains, and dairy products, and greater consumption of energy-dense non-nutrient foods. This was also associated with lower income and housing inadequacy.
Johnson-Down & Egeland, 2010 [30]	Behaviour-Dietary	388 participants; 16 communities	3–5	Male: 47 %; Female: 53 %	Nunavut	Quantitative/Cross-sectional	Most of the children met their dietary requirements of energy and micro nutrients through consumption of TFs. The findings further showed that energy-dense foods and beverages contributed significantly to their diets and placed the children at increased risk of overweight, obesity, and tooth decay.
Kolahdooz et al., 2013 [59]	Determinant-Smoking	92 participants; 3 communities	19–44	Female: 100 %	Inuvialuit	Quantitative/Cross-sectional	No significant differences were observed in nutrients intakes between smokers and non-smokers. Regardless of their smoking status, over 60 % of respondents did not meet their daily recommendations for fibre, vitamins D, E, and Potassium.
Kolahdooz *et al*., 2013 [60]	Determinant-Drinking	92 participants; 3 communities	19–44	Female: 100 %	Inuvialuit	Quantitative/Cross-sectional	Energy consumption was significantly higher among drinkers in comparison to non-drinkers. Although there were no significant differences in most nutrients intakes between drinkers and non-drinkers, drinkers tended to have decreased nutrient density compared to non-drinkers.
Kuhnlein et al., 1996 [29]	Behaviour-Dietary	366 participants; 1 community	≥ 3	Not reported	Nunavut	Quantitative/Cross-sectional	TFs provided significantly higher levels of protein and micro nutrients, and less energy and carbohydrates for most age groups than store-bought foods. There was a significant seasonal variation in the consumption of TFs in contrast to store-bought foods.
Kuhnlein *et al*. 2004 [16]	Behaviour-Dietary	3851 participants: 44 communities	≥ 13	Not reported	Yukon, NWT & Nunavut	Quantitative/Cross-sectional	TF intake was associated with lower fat, carbohydrate, and sugar, greater protein, vitamins and most micronutrients, in the diet. Adults 40 years and over had significantly higher intakes of TFs compared to younger respondents.
Kuhnlein & Receveur, 2007 [28]	Behaviour-Dietary	3851 participants; 44 communities	≥ 13	Not reported	Yukon, NWT, & Nunavut	Quantitative/Cross-sectional	TFs contributed about 6–40 % of energy among adults compared to 0.4–15 % among children. Greater amount of energy was contributed by sugar-sweetened beverages and other energy-dense foods in children diets.
Lambden *et al*., 2006 [50]	Determinant-Socioeconomic factors	1711: 838 Inuit; 511 Dene/Métis; 422 First Nation; participants; 44 communities	≥ 20	Female: 100 %	Canadian Arctic	^a^Mixed/Cross-sectional	There were significant regional variations across communities in terms of affordability of foods, ranging from 40 to 70 %. These variations were also reflected on other measures such as accessibility and affordability of hunting/fishing equipment; significant proportion of respondents could not afford hunting/fishing equipment.
Lambden *et al*., 2007 [42]	Determinant-Historical and cultural factors	1711: 838 Inuit; 422 Yukon First Nations; 511 Dene/Métis; 44 communities	≥ 20	Female: 100 %	Yukon & NWT	^a^Mixed/Cross-sectional	Although TFs are emblematic of cultural identity, and are socially well received, the quality of many local food species has however deteriorated in the last few decades.
Lardeau *et al*., 2011 [54]	Determinant-Socioeconomic factors	8 participants; 1 community	≥18	37.5 % male; 62.5 % female	Nunavut	Qualitative/Cross-sectional	Affordability was a major factor influencing food security in Iqaluit. Community members with low SES relied on social support networks to meet their basic dietary needs.
Martin, 2011 [43]	Determinant-Historical and cultural factors	24 participants; 1 community	≥ 16	Male: 46 %; Female: 54 %	Nunatsiavut	Qualitative/Cross-sectional	Dietary transitions from locally sourced, unstable food environment to contemporary times that presented a choice between healthy and unhealthy store-bought foods posed a challenge to residents. TF gathering activity was identified as an opportunity for physical activity.
Mead *et al*., 2010 [41]	Determinant-Historical and cultural factors	43 participants; 2 communities	≥19	14 % male: 84 % female	Nunavut	Qualitative/Cross-sectional	Dietary transition was due to changes from traditional ways of life to Euro-Canadian lifestyles. Although TFs were perceived as healthier than store-bought foods, high cost of hunting materials affected the availability of TFs. Cost was also a major barrier of access to healthy store-bought foods, while transportation and harsh climate hindered access to fruits and vegetables.
Mead *et al*., 2010 [55]	Determinant-Psychosocial factors	266 participants; 3 communities	41.2 ± 13.6	Not reported	Nunavut	Quantitative/Cross-sectional	Greater knowledge about healthy foods and self-efficacy were associated with intentions toward healthy food consumption. . Self-efficacy was associated with decreased acquisition of unhealthy foods and increased acceptance of healthier food preparation methods. Additionally, SES was positively correlated with healthy food knowledge, acquisition, and preparation behaviors.
Mead *et al*., 2010 [56]	Determinant-Psychosocial factors	231 participants; 3 communities	43.4 ± 13.6	Not reported	Inuvialuit	Quantitative/Cross-sectional	Greater intention toward healthy food consumption was positively correlated with increased frequency of healthy food acquisition and decreased frequency of unhealthy food acquisition. The choice of healthier food preparation methods was associated with knowledge of healthy foods, intentions, and self-efficacy.
Mead *et al*., 2012 [61]	Determinant-Intervention	379 participants; 6 communities	Male: 42.4 ± 13.1; Female: 42.3 ± 12.8	Male: 18 %; Female: 82 %	Nunavut & Inuvialuit	Quantitative/Intervention	Respondents from intervention communities demonstrated greater food-related self-efficacy and intentions compared to respondents from control communities. Over-weight, obese, and higher SES respondents demonstrated greater improvements compared to control.
Nancarrow & Chan, 2010 [45]	Determinant-Climatic factors	17 participants; 2 communities	≥ 18	Male: 76 %; Female: 24 %	Nunavut	Qualitative/Cross-sectional	Climate change had both positive and negative effects on accessibility and availability of TF species.
Rittmueller *et al*., 2012 [57]	Determinant-smoking	218 participants; 3 communities	19–79	21 % male; 79 % female	Inuvialuit	Quantitative/Cross-sectional	Both male and female smokers reported higher intakes of energy and some other nutrients compared to non-smokers. However, more than 50 % of both male and female smokers had insufficient intakes of fibre, Potassium, and vitamin E. Additionally, TFs contributed about 3–6 % less energy and protein intakes among smokers compared to non-smokers.
Rittmueller *et al*., 2012 [58]	Determinant-Smoking	208 participants; 3 communities	19–79	15 % male; 85 % female	Nunavut	Quantitative/Cross-sectional	Smokers were likely to consume lower amounts of nutrient-dense TFs but higher energy-dense foods, compared to non-smokers, suggesting increased dietary inadequacies among smokers.
Rode & Sheppard, 1984 [36]	Behaviour-Physical activity	344 participants; 1 community	Male: 9–76	Male: 58.4 %; Female: 41.6 %	Nunavut	Quantitative/Longitudinal	There was a decreased fitness level in the population determined by a 15 % decrease in predicted maximum oxygen intake, a 2–4 kg rise in BMI, build up of subcutaneous fat, and reduced leg extension strength in all age groups except 9–15 years old, in comparison to the 1970–71 data.
Rode & Sheppard, 1994 [37]	Behaviour-Physical activity	221 participants; 1 community	20–69	Male: 57.5 %; Female: 42.5 %	Nunavut	Quantitative/Longitudinal	Fitness levels had remarkably deteriorated over a 20-year period (1970–1990). However, community members who actively engaged in regular sports had maintained their fitness at levels observed in the 1970s, based on data comparison.
Rosol *et al*., 2011 [32]	Behaviour-Dietary	2595 participants; 36 communities	≥ 18	Not reported	Inuvialuit; Nunatsiavut & Nunavut	Quantitative/ Cross-sectional	The severity of food insecurity differed across the three regions of study. Nunavut had the highest prevalence at 68.8 %, followed by Nunatsiavut and Inuvialuit regions at 45.7 % and 43.3 %, respectively.
Sharma *et al*., 2009 [34]	Behaviour-Dietary	101 participants; 2 communities	≥ 19	47.5 % male; 52.5 % female	Inuvialuit	Quantitative/Cross-sectional	Dietary intakes of fibre and most micronutrients were lower than requirements. Less nutrient-dense, store-bought foods were the most frequently consumed food items. Among these, sugar and sugar-sweetened beverages were the leading contributors to energy intake.
Sharma *et al*., 2010 [35]	Behaviour-Dietary	87 participants; 2 communities	19–87	47 % male; 53 % female	Nunavut	Quantitative/Cross-sectional	Dietary intakes of fibre and most micronutrients were significantly below recommendations. Less nutrient-dense store-bought foods were more frequently consumed than nutrient-rich TFs.
Sharma *et al*., 2013 [1]	Behaviour-Dietary	211 participants; 3 communities	Male: 42.4 ± 13.2; Female: 42.1 ± 15	Male: 17 %; Female; 83 %	Nunavut	Quantitative/Cross-sectional	Less than 10 % of respondents met their dietary requirements. 22 % of saturated fat, 30 % of energy, and 73 % of sugar came from non-nutrient dense foods, while TFs contributed 49 % of Iron and 56 % of protein intake among women.
Sheehy *et al*., 2013 [11]	Behaviour-Dietary	211 participants; 3 communities	Male: 42.4 ± 13.2; Female: 42.1 ± 15	Male: 17 %; Female; 83 %	Nunavut	Quantitative/Cross-sectional	TFs including caribou, muktuk and arctic char were widely consumed. Additionally, sugar-sweetened beverages and other energy-dense foods were consumed in significant amounts as of the time of study compared to the past.
Sheikh *et al*., 2011 [10]	Behaviour-Dietary	2595 participants; 36 communities	41 ± 14.7	Male: 38 %; Female: 62 %	Inuvialuit, Nunavut & Nunatsiavut	Quantitative/Longitudinal	Contribution to energy from TFs had significantly decreased over the ten year period, while consumption of store-bought foods rose remarkably. BMI also significantly increased over the period, particularly for women.
Wein & Freeman, 1992 [26]	Behaviour-Dietary	71 participants; 1 community	≥10	Not reported	Inuvialuit & NWT	Quantitative/Cross-sectional	Climate change was associated with lower TF availability and use. This resulted to reduced intakes of nutrients normally sourced from TFs.
Wein *et al*., 1996 [27]	Behaviour-Dietary	164 participants; 1 community	≥12	Not reported	Nunavut	Quantitative/Cross-sectional	TFs were preferred, rated high, and consumed by majority of adults and young people. However, from a total of 41 foods, adults ranked 25 TFs higher and two store-bought foods lower than young people using a five point hedonic scale.
Wesche & Chan, 2010 [18]	Determinant-Climatic factors	30 communities; ample size not reported	≥15	Not reported	Inuvialuit, Nunavut, Nunavik & Nunatsiavut	Qualitative/Cross-sectional	TF availability was influenced differentially across the communities studied by factors including impact of climate change, harvesting patterns, individual species reliability, availability and access to other food species.
Zotor *et al*., 2012 [12]	Behaviour-Dietary	230 participants; 3 communities	Male:44 ± 14; Female: 41 ± 13	Male: 24 %; Female: 76 %	NWT	Quantitative/Cross-sectional	Non-nutrient dense foods were consumed at significantly higher frequencies per day, compared to TFs, fruits, and vegetables.

^a^refers to a combination of quantitative and qualitative methodologies

### Behavior articles

#### Dietary

##### Age- and gender-related consumption of traditional versus store-bought foods

Patterns of dietary behavior have been studied across geographic (Canadian Inuit) regions and distinct demographic (gender, age) factors, 10 articles [10–12, 16, 25–30] reported on the consumption patterns of TFs versus store-bought foods. A longitudinal study [10] in 18 Inuit communities reported a significant decrease in TF contribution to energy intake compared to store-bought foods between 1999 and 2008. Older adults consistently consumed more TFs than younger people while women consumed less TFs than men, irrespective of age [10]. Additionally, cross-sectional studies in Nunavut [11], Inuvialuit [12], and Nunavik [25] reported moderate decline in consumption of TFs and increased intakes of non-nutrient energy-dense store-bought foods. The results were attributed to the shifting dietary patterns from subsistence living to wage economy, the high cost of hunting equipment, and reduced TF sharing practices. Further, an Inuvialuit study [26] determined annual consumption frequencies of 32 species of mammal, fish, bird, and plant by adults and children. The article reported that TFs were significantly less consumed than store-bought foods. Although there were no significant differences in the children’s preferential ranking of 31 out of 34 TFs when compared to adults’ preferences, children however rated store-bought foods higher than adults. This result was similar to that obtained among Belcher Island Inuit of Nunavut [27].

Five other articles described age-and-gender-related consumption patterns [16, 20, 28–30]. Kuhnlein and colleagues found that the consumption of TFs by older adults was greater than intakes by younger adults and children. Consumption of TFs was generally low for all ages compared to pre-contact era when TFs were the only source of foods [16]. Contribution to energy from TFs range from 6 to 40 % among adults compared to 0.4–15 % among children, while more than 40 % of children’s daily energy was sourced from non-nutrient energy-dense store-bought foods [28]. Similarly, results from an earlier study showed that TF consumption by older Inuit was higher than among younger Inuit, and that store-bought foods contributed significantly more to carbohydrates and saturated fat intakes among people less than 60 years old, while TF contributed more proteins and micronutrients for all ages (except for children and teenagers) and for both genders [29]. Furthermore, a cross-sectional study [30] that examined TF consumption patterns of three to five year old Inuit children in16 communities reported that a majority of children met the dietary requirements of most nutrients, although less than 50 % of the children met the fibre requirement. Significant portions of vitamins and micronutrients were generally derived from TF consumption. The findings show that both age and gender are important factors influencing consumptions of both TFs and store-bought foods among Inuit.

#### Dietary inadequacies

Eight articles [1, 13, 20, 31–35] assessed geographically determined dietary patterns by quantifying the prevalence of dietary inadequacies among various Canadian Inuit populations. The first three articles reported high prevalence of dietary inadequacies in Nunavut [13] and Inuvialuit [1, 31] regions. Food insecurity was also assessed in 36 communities spread across three Inuit regions: Nunavut, Nunatsiavut, and Inuvialuit [32]. The results indicated that Nunavut has the highest rate of food inadequacy at 68.8 %, which was significantly higher than Nunatsiavut and Inuvialuit regions at 45.7 % and 43.3 %, respectively [32]. The pervasive degree of food insecurity in Nunavut was also evident among Inuit children [33]. According to Egeland and colleagues, approximately 70 % of the children resided in food insecure households: 31 % of the children were moderately food insecure; 25.1 % experienced severe food insecurity. Two other articles that described dietary sufficiency as a measure of household food security reported that consumptions of fibre, essential vitamins and micronutrients were significantly lower than recommended levels in Inuvialuit [34] and Nunavut [35] communities studied. Less nutritious energy-dense store-bought foods were heavily consumed both in frequency and quantity by participants in both regions. In terms of gender-related differences, food insecurity was correlated with reduced intake of energy, fibre, Iron, Magnesium, Zinc and vitamin C among men. In women, food insecurity was correlated with higher intake of carbohydrate and lower intake of fibre, folate, vitamins C, and D, Magnesium and Calcium. TF consumption was associated with higher intakes of protein, vitamins A and C, lower intakes of carbohydrate, saturated fat, fibre, and Sodium [20]. These findings suggest strong relationships between dietary behaviors (food choices and consumption patterns) and the demographic factors.

#### Physical activity and fitness

Five articles [36–40] described the fitness and physical activity levels of Canadian Inuit. Of these, two articles reported on a longitudinal study [36, 37], the next two articles [38, 39] were published on a cross-sectional study conducted in two Inuit regions while the fifth article reported on a multi-jurisdictional study that included other Indigenous groups [40]. In the first longitudinal study of Rode and Sheppard [36] changes in fitness levels in a Nunavut community were examined, They found that from 1969 to 1982, and for all ages and genders (except boys 9–15 years), there was a 15 % decrease in predicted maximum oxygen intake, two to four kilogram increase in body weight, an accumulation of subcutaneous fat, a loss of lean muscles, and a decrease in leg extension strength (a measure of lower body strength and fitness).

Twenty years after the 1969/1970 study, Rode and Sheppard conducted a second follow-up assessment of the fitness levels in the face of rapid acculturation process in Nunavut [37]. Longitudinal comparisons with the 1969/70 study showed decreases in physical activities, aerobic power and muscle strength, and increases in subcutaneous fats. The BMI of younger men were lower in 1989/1990 compared to the 1969/70, but significantly higher in men 40 years and above. In women, there was a slight difference for those under 40 years while the BMI was higher for women 40 years and over [37]. Two more recent articles [38, 39] described the levels of physical activity and BMI in three Nunavut communities [38] and three Inuvialuit communities [39]. According to the findings, about 89 % of respondents self-reported medium to high levels of physical activity in both regions. Despite this, approximately 72 % and 65 % of Nunavut and Inuvialuit research subjects, respectively, were either overweight or obese, with more women in the obese category than men. There is therefore a co-existence of overweight or obesity and high levels of physical activity among the Inuit. These outcomes contradicted results from a more recent survey by Statistics Canada [40] which reported that only 31 % of Inuit were physically active at leisure time, contradicting the results obtained by Hopping et al. in which 89 % of respondents self-reported medium to high levels of physical activity [38, 39]. The inconsistencies in the findings call for new studies that utilize objective methodology for assessing physical activity levels in the population.

### Determinant articles

#### Historical and cultural factors

Three articles [41–43] reported on historical and cultural determinants of dietary behaviours and physical activity. In their cross-sectional study, Mead and colleagues [41] examined the factors influencing changing food environment and contemporary dietary practices of Inuit in two communities in Nunavut. The findings showed that, although Inuit cultural values are still being upheld in some communities today, generally, many aspects of Inuit cultural traditions have been eroded, such as hunting and food sharing practices, by the domineering influence of Euro-Canadian lifestyles, particularly among the younger generation [41]. Over the past five decades, Inuit have been increasingly consuming store-bought foods at the expense of TFs. This reflects a changing food environment and erosion of cultural traditions [41]. Other researchers [42, 43] emphasized the inextricable linkage between TFs and culture among Inuit. Findings from a cross-sectional study by Lambden and colleagues [42] among Yukon First Nation, Dene/metis, and Inuit women in 44 Arctic communities buttressed the notion that TFs are healthy, and are socially and culturally beneficial but the quality has deteriorated over time. Martin [43] reported that traditional practices around food should be seen as “symbolic” of Inuit cultural identity. Underscoring the importance of food as a cultural construction among the Inuit, Meigs [44] explained that certain cultural and social meanings are attributable to food sharing practices by Inuit, and traditional foods consist mainly of land and marine animals. The TFs are a marker of traditional and social practices which are embedded in Inuit cultural identity [44]. Thus, over several decades colonization and globalization have profoundly contributed to the erosion of Inuit cultural traditions including the traditional food gathering, consumption, and sharing practices.

#### Climatic factors

Two articles [18, 45] described the impact of the physical environment such as climate change on diet selection. Wesche and Chan [18] conducted a regional case study analysis with Inuvialuit elders to determine the impact of climate change on nutritional health. The study showed that virtually all aspects of species abundance, migration patterns and wellbeing are affected by climate change. Hunter-gatherer movement patterns had equally changed in response to food species migratory patterns which were reportedly influenced by climate change [18]. In another cross-sectional study [45], bivalent effects of environmental changes were observed, ranging from migratory patterns of land and marine animals, to quality and availability of food animal species. Although no consistent trends were observed in the study, many aspects of the findings were corroborated by Wesche and Chan [18]. Despite the ambivalence about the impacts of climate change in the Arctic, it is increasingly becoming evident that both availability and quality of some food species are being affected.

#### Socioeconomic factors

A total of nine articles [46–54] described the influence of education and income on consumption patterns of Canadian Inuit. In Nunavut [46] and Inuvialuit [47], Hopping and colleagues found a positive correlation between education attainment and higher income with increased frequency of fruit and vegetable consumption. Three articles [48, 49, 51] reported on the livelihood challenges facing Canadian Inuit in the face of rapid socioeconomic and climate changes in the Arctic. In the first two articles [48, 49] research participants in Igloolik community in Nunavut identified socioeconomic factors that include availability and affordability, high cost of harvesting TFs, poor budgeting skills, low education and poor knowledge of nutrition, as challenges to accessing healthy foods. These results were corroborated by earlier findings by Lambden *et al*. [50], which noted further that the extent of the problem varied across communities [50].

In a related study, Ford and colleagues [51] characterized users of community food programs in Iqaluit and determined that the individuals were socioeconomically disadvantaged. Additionally, two other articles reported on an assessment of the relationship between SES and dietary patterns in three Inuit regions: Inuvialuit, Nunavut and Nunatsiavut [52, 54]. Huet and colleagues [52] found that food insecure households had lower healthy eating index score, consumed less fruits, vegetables, grains, and dairy products but had significantly more non-nutrient energy dense foods, compared to those from food secure households. These individuals were likely to live on income support, overcrowded homes and houses in need of major repairs, compared to individuals from food secure homes. Lower SES had a stronger impact on food security in households with children [53]. As well, participants in a Photovoice study [54] identified high costs and limited choice of healthy foods, poor budgeting skills, and addictions as factors aggravating food insecurity among low SES residents of Iqaluit community in Nunavut. These findings generally underscore the critical role of socioeconomic factors of education and income on energy balance-related behaviors of Canadian Inuit.

#### Psychosocial factors

Two articles [55, 56] described the mediating role of psychosocial factors on dietary behavior and physical activity. Mead and colleagues examined the relationship between food acquisition and preparation behaviors of Inuit in Nunavut [55] and Inuvialuit [56] and the psychosocial and socioeconomic factors influencing these behaviors. According to findings from both regions, intention was positively associated with the frequency of healthy foods acquisition. Further, there was an association between the use of food preparation practices that were considered healthier and increased healthy foods knowledge, intention, and self-efficacy. Higher level of education was positively correlated with increased healthy food self-efficacy. Increased healthy food knowledge and self-efficacy were also associated with greater intentions to make healthier food choices. This suggests potential roles for psychosocial factors in the regulation of energy balance-related behaviors of Canadian Inuit.

#### Smoking and drinking

Four articles [57–60] described the impact of smoking and drinking behaviors on dietary adequacy of Canadian Inuit. One cross-sectional study examined the association between smoking status and dietary adequacy among Inuit in three Inuvialuit communities [57] and three Nunavut communities based on smoking status [58]. In another cross-sectional study, the influence of smoking status [59] and alcohol consumption [60] was examined. Rittmueller and colleagues found that smokers tend to consume more energy-dense and less nutritious foods compared to non-smokers, while the study conducted specifically on Inuvialuit women [59] demonstrated that smokers were likely to be deficient in vitamin C. The article by Kolahdooz and colleagues [60] on the impact of alcohol consumption on dietary adequacy reported that alcohol consumption altered nutrient intakes of Inuvialuit women of child bearing age, and that energy intakes of drinkers were higher than non-drinkers, but no differences in nutrient intakes between drinkers and non-drinkers were found except that drinkers had lesser nutrient density [60]. Therefore it appears some unhealthy behaviors including smoking and drinking may adversely influence eating habits and diet qualities in the population.

#### Health promotion intervention articles

Two articles [61, 62] reported on health intervention programs designed to promote dietary behaviors and/or physical activity amongst Canadian Inuit. One article [61] reported on health promotion intervention programs that incorporated some psychosocial constructs to address the growing burden of chronic diseases in Nunavut and Inuvialuit. The nutrition and lifestyle intervention program (Healthy Foods North) was underpinned by social cognitive theories of human behavior and implemented in multiple environmental settings such as schools and recreational centers. Pre- and post-evaluation of the intervention program showed an increase in food-related self-efficacy and intentions in intervention communities, compared to participants from comparison communities [61]. Additionally, participants who were overweight, obese and had higher SES displayed more improvements in the constructs examined, such as self-efficacy, healthy eating knowledge and behavioral interventions, compared to those with lower SES or those within healthy weights range [61]. Another intervention program that was designed for Nunavik children who attended day care centers was reported to have improved the nutrition status of participating children [62]. According to the findings, the proportion of children who met the recommended servings of fruits, vegetables, and grains, as well as requirements of vitamins, irons, and other micronutrients through the nutrition intervention program was significantly greater than those who did not participate in the program [62]. Although these studies are limited, the findings on the sociocogntive constructs are promising and present opportunities for further intervention research.

## Discussion

The purpose of this study was to identify cultural, environmental, socioeconomic and psychosocial determinants of energy balance-related behaviors among Canadian Inuit. Our main findings indicate that sociocultural and environmental changes are responsible for transitions from healthy traditional diets to less nutrient energy-dense store-bought foods in Inuit communities. We also documented a shift in lifestyle among Inuit from an active hunter-gatherer subsistence living to a more sedentary and motorized lifestyle over the last 50 years. In this section, we discussed the various factors influencing energy balance-related behaviors in Inuit communities and identified areas where additional research is required to foster our understanding of determinants of these behaviors.

Our findings showed that the social and cultural changes that swept across Inuit communities since the first contact with European immigrants have profoundly impacted virtually all spheres of Inuit life, which historically was rooted in Indigenous traditions. It was also evident from this review that many aspects of Inuit traditional life have been eroded, particularly the hunter-gatherer identity and traditional food sharing practices. Food gathering and sharing are essential traditional activities and are both symbolic of Inuit cultural identity [63]. However, Euro-Canadian lifestyles and its domineering influence have to a large extent subjugated Inuit cultural traditions, and today, has profoundly influenced the energy balance-related behaviors. Apart from increasingly becoming a less active population in contrast to pre-contact era, there is a growing interest in less nutrient energy-dense store-bought Euro-Canadian diets among the younger generation of Inuit. This has in the last five decades resulted to weight gain, with increasing concerns about obesity trends in the population particularly amongst children. Additionally, a large proportion of Inuit are of lower socioeconomic status (SES), measured by education and income, which further aggravates the problem. A lower SES status and pervasive household food inadequacy substantially reduce the ability to access healthy foods and perform healthy dietary behaviors in the face of limited affordable choices. All these suggest that policy makers and health interventionists must begin to develop programs and adaptation mechanisms to accommodate the social, cultural and environmental realities of the population.

In the last five decades increasingly less traditional food gathering and culture-based recreational activities have taken place compared to pre-contact era when all foods were locally sourced through hunting, trapping, and fishing. Although TFs are culturally preferred and more nutritious than store-bought foods, studies conducted on the changing dietary patterns of Inuit strongly suggest decreasing contributions of TFs to total energy and nutrient intakes in terms of frequency and quantity, particularly among younger people. The decreasing contributions by TF are attributable to a number of factors. These include the rapidly growing population rate, and increasing population of younger generation with limited hunting skills and growing dependence on wage-based economy, in which individuals earn income from paid employment reducing the need and possibility of actively engaging in TF harvesting and processing [16, 20].

Additionally, climate change and the environmental impacts on both flora and fauna were also documented in this review. Increase in environmental temperatures and changes to the migratory patterns of both terrestrial and sea mammals have all combined to reduce accessibility and availability of TFs [64, 65]. The implication is that Inuit are compelled to be less dependent on TFs, and are increasingly becoming reliant on non-traditional and often less healthy store-bought foods “imported” from Southern Canada, as popular alternatives. The dietary inadequacies among Canadian Inuit are also well documented. At prevalence rates ranging from 43.3 to 68.8 % [32], food insecurity is at a level that can be described as a public health emergency. A large percentage of Inuit population struggle to access adequate nutritious foods. The rate of food insecurity in the region is described as highest of any Indigenous population groups in North America [33].

Published articles that reported on determinants of physical (in)activity in Canadian Inuit population are relatively scarce. It is crystal clear from the longitudinal studies that a change from hunting-related subsistence ways of life to wage-based economy, mechanized and sedentary living, due to Euro-Canadian cultural influences, were responsible for lower rates of participation in physical activity and corresponding fitness levels. Published results of the more recent studies on physical activities are inconsistent and perhaps unreliable because, despite claims that self-report measures have been validated in this culture, the instruments appear to be questionable measures for determining physical activity levels in Inuit population. For example, such instruments may not fully account for measuring physical activity levels of Inuit who engage in traditional and often seasonal “on-the-land activities”. The inconsistencies observed in reported findings [38–40] suggest that more research is needed to determine reliable age-specific physical activity levels in the population. Articles by Hopping et al. [38, 39] reported a co-existence of overweight/obesity and high levels of physical activity in both Nunavut and Inuvialuit. This suggests that the use of IPAQ instrument was not appropriate for Inuit. A more objective measure of physical activity, such as accelerometry, might produce better results for the population as was the case for Greenland Inuit. Accelerometry in combination with heart rate measurement produced more accurate assessments of physical activity amongst Greenland Inuit [66, 67], and when contrasted with IPAQ, generated more reliable results [66].

There is a general paucity of published articles on physical (in)activity of Canadian Inuit to inform health promotion intervention planning and decision making. To our knowledge, no published articles have explored the socio-economic influences on physical activity-related behaviors, and socioeconomic differences in perceived barriers to physical activity and potential individual, household, community, as well as environmental and policy determinants of these differences. Such information is critical in order to understand upstream factors influencing the environments (built, social, community infrastructure, policy, etc.) and the impacts on leisure-time physical activity among Canadian Inuit. Such information is needed to inform the systematic theory- and evidence based development of interventions in the population.

A reasonable body of evidence in Nunavut and Inuvialuit suggests influencing roles for SES, measured by income and education, as well as sociodemographic factors of age and gender on dietary behaviors of Inuit. Findings from studies in both Nunavut and Inuvialuit showed that Inuit households with low education and income, and poor nutrition education, are more likely to consume less fruits and vegetables in terms of frequency and quantity, but more energy-dense store-bought foods [50–53]. Additionally, children and younger adults below the age of 50 were more likely to consume more fruits and vegetables, less TFs and less nutrient-dense foods, compared to older Inuit [46, 47]. Gender differences were less significant [20, 31]. Such information on demographic differences can be used to target interventions to population groups, especially based on age, income and education, which may benefit more from intervention programs.

In the context of health promotion, two intervention studies were developed based on social cognitive theories of human behavior and that incorporated some environmental determinants of energy balance-related behaviors. These studies showed some positive changes in energy balance-related behaviors among Canadian Inuit [62, 63], but it remains unclear whether these changes were sustained over time. Additional studies are therefore needed to deepen our understanding of cognitive and environmental factors influencing energy balance-related behaviors among Canadian Inuit.

## Conclusion/Recommendation

Canadian Inuit have undergone significant environmental, cultural and social changes that have eroded the cultural values and indigenous ways of life leading to very limited traditional resources to support healthy lifestyle. Rapid westernization and globalization due to colonization, as well as environmental transitions due to climate change, seem to be the main causes of these changes observed. The changes have reduced the reliance on TF gathering and processing activities and increased dependence on energy-dense store-bought foods and motorized transportation. Although these environmental and social changes cannot be reversed or stopped, opportunities exist to explore behavioral change models and policy interventions for health promotion in Inuit population. Effective health promotion interventions for Inuit population are at present very limited or non-existing. Therefore, we recommend that future research focus on examining how energy balance-related behaviors at individual, household and community levels in the Canadian Arctic can be influenced to promote health and reduce chronic disease burden on the population.

## Appendix A

*Search terms string 1:* Inuit OR Canadian Inuit OR Arctic Inuit OR Canadian Eskimo OR Nunaat OR Inuvialuit OR Nunavik OR Nunatsiavut OR Nunavut.

AND

*Search terms string 2:* Physical activity OR exercise OR fitness OR active life OR active OR dancing OR singing, OR hunting OR fishing OR determinant of exercise OR determinant of obesity OR determinant of overweight OR determinant of chronic disease OR determinant of fitness OR determinant of diet OR food gatherer OR food gatherers OR traditional games OR games OR cultural activity OR cultural activities OR traditional activity OR traditional activities OR food gathering OR diet OR dietary OR diet selection OR food habit OR food habits OR eating, eating habit, eating habits, eating behavior, eating behaviors, food, junk food, junk foods, feeding behavior RO feeding behaviors OR traditional food OR country food OR cultural food OR local food OR local foods OR meal OR meals OR meal pattern OR meal patterns OR micronutrient OR micronutrients OR macronutrient OR macronutrients OR sodium chloride OR minerals OR drinking OR snacking OR fruit OR fruits, OR vegetable OR vegetables OR berries OR sugar OR energy-dense OR fat OR fatty OR protein OR carbohydrate OR minerals OR seal OR whale OR fish OR arctic char OR caribou OR vitamin OR vitamins.
